# Coloured Rice Phenolic Extracts Increase Expression of Genes Associated with Insulin Secretion in Rat Pancreatic Insulinoma β-cells

**DOI:** 10.3390/ijms21093314

**Published:** 2020-05-07

**Authors:** Gideon Gatluak Kang, Nidhish Francis, Rodney Hill, Daniel LE Waters, Christopher L. Blanchard, Abishek Bommannan Santhakumar

**Affiliations:** 1 Australian Research Council (ARC) Industrial Transformation Training Centre (ITTC) for Functional Grains, Graham Centre for Agricultural Innovation, Wagga Wagga, NSW 2650, Australia; gkang@csu.edu.au (G.G.K.); nfrancis@csu.edu.au (N.F.); dawaters@csu.edu.au (D.L.E.W.); cblanchard@csu.edu.au (C.L.B.); 2 School of Biomedical Sciences, Charles Sturt University, NSW 2650, Australia; rhill@csu.edu.au; 3 School of Animal and Veterinary Sciences, Charles Sturt University, NSW 2650, Australia

**Keywords:** coloured rice, polyphenols, gene expression, pancreatic β-cells function, insulin secretion, type 2 diabetes

## Abstract

Glucose-induced oxidative stress is associated with the overproduction of reactive oxygen species (ROS), which may dysregulate the expression of genes controlling insulin secretion leading to β-cell dysfunction, a hallmark of type 2 diabetes mellitus (T2DM). This study investigated the impact of coloured rice phenolic extracts (CRPEs) on the expression of key genes associated with β-cell function in pancreatic β-cells (INS-1E). These genes included glucose transporter 2 (*Glut2*)*,* silent mating type information regulation 2 homolog 1 (*Sirt1*)*,* mitochondrial transcription factor A (*Tfam*)*,* pancreatic/duodenal homeobox protein 1 (*Pdx-1*) and insulin 1 (*Ins1*). INS-1E cells were cultured in high glucose (25 mM) to induce glucotoxic stress conditions (HGSC) and in normal glucose conditions (NGC-11.1 mM) to represent normal β-cell function. Cells were treated with CRPEs derived from two coloured rice cultivars, Purple and Yunlu29-red varieties at concentrations ranged from 50 to 250 µg/mL. CRPEs upregulated the expression of *Glut2, Sirt1* and *Pdx-1* significantly at 250 µg/mL under HGSC. CRPEs from both cultivars also upregulated *Glut2, Sirt1, Tfam, Pdx-1* and *Ins1* markedly at 250 µg/mL under NGC with Yunlu29 having the greatest effect. These data suggest that CRPEs may reduce β-cell dysfunction in T2DM by upregulating the expression of genes involved in insulin secretion pathways.

## 1. Introduction

Pancreatic β-cells are the major sites for synthesis, storage and secretion of insulin and amylin, hormones that regulate blood glucose levels. Correct β-cell function is indispensable for blood glucohomeostasis and the management of type 1 and type 2 diabetes [1]. However, pancreatic β-cell dysfunction is believed to be a major contributing factor to the pathogenesis of diabetes. Chronic high glucose levels (hyperglycaemia) have been associated with oxidative stress leading to the overproduction of reactive oxygen species (ROS) including superoxide (O2^−^), hydroxyl radical (.OH), hydroxyl ion (OH^−^) and hydrogen peroxide (H_2_O_2_) [2]. High glucose-induced oxidative stress has been linked with significant damage to cellular molecules including DNA and proteins thereby disrupting cellular signalling and resulting in dysregulation of various genes associated with insulin secretion and β-cell function [3]. 

The processes leading to insulin release involve the initial glucose entry into β-cells followed by mitochondrial adenosine triphosphate (ATP) generation and K^+^/Ca^2+^ membrane depolarisation leading to exocytosis events. These pathways are regulated by some key genes such as *Glut2*, *Sirt1*, *Tfam*, *Pdx-1* and *Ins1*. Glucose is transported into pancreatic β-cells via *Glut2*, a high-Km glucose transporter serving as a “gatekeeper” for β-cells metabolic signalling, and converted to pyruvate [4]. High glucose-induced stress has been shown to reduce the expression of *Glut2*, severely compromising glucose sensing mechanisms which leads to β-cell dysfunction. 

In mitochondria, *Sirt1*, a member of nicotinamide adenine dinucleotide (NAD)-dependent histone deacetylases, serves as a key energy redox sensor which regulates the NAD^+^/NADH ratio to generate ATP [5]. Thus, an important function of *Sirt1* is associated with glucose homeostasis and mitochondrial metabolism in glucose sensitive tissues such as β-cells [6]. Previous studies have demonstrated that *Sirt1* was down regulated in diabetic subjects, which was concomitantly linked to the reduction in mitochondrial membrane hyperpolarisation and oxygen consumption leading to an impaired β-cell function [7,8]. 

*Tfam* is a nuclear-encoded transcription factor that controls the stability and transcriptional activity of mitochondrial DNA (mtDNA). Coupled with electron transport chain regulation, *Tfam* is also involved in insulin exocytosis events by maintaining the ADP/ATP ratio [9]. The resulting K^+^ channel inactivation and Ca^2+^ influx triggers insulin secretion, thereby enhancing β-cell function. It has been reported that the expression of *Tfam* was downregulated under HGSC in human islets, which reduced mtDNA stability and β-cell function [10]. 

*Pdx-1* is considered to be a master β-cell regulatory gene that controls major transcriptional β-cell insulin secretion mechanisms, thus playing an important role in mitochondrial embryonic development and β-cell differentiation [11]. Upregulation of *Pdx-1* has been shown to enhance metabolic pathways driving glucose stimulated insulin secretion (GSIS) and β-cell function [12]. A study revealed that high glucose-induced oxidative stress downregulated *Pdx-1,* which correlated with β-cell failure and the development of T2DM in animal models [13]. *Ins1* encodes the hormone insulin and plays a vital role in insulin biosynthesis and insulin secretion. An early investigation demonstrated that prolonged exposure of β-cells to high glucose levels is linked to decreased proinsulin biosynthesis and the downregulation of *Ins1* [14]. 

Plant-based diets possess antioxidant properties and recent attention has focused on their activities to alleviate the pathogenesis of many diseases including T2DM. Several studies have demonstrated that consumption of polyphenolic-rich food may protect β-cells from oxidative damage and ameliorate β-cells dysfunction by modulating the expression of genes involved in insulin secretion pathways [15,16]. Coloured rice varieties, including Purple and Yunlu29, have antioxidant properties [17] which have been associated with blood glucose lowering effects in diabetic models [18,19]. Phenolic extracts, from various dietary sources, containing phenolic compounds cynidin-3-*0*-glucoside, catechins and gallic acids upregulate β-cell function genes which are correlated with improved insulin secretion and reduced high blood glucose levels [20,21,22,23,24,25]. These phenolic compounds are also present in varying amounts in coloured rice varieties. However, it is yet to be elucidated whether CRPEs can modulate the expression of genes involved in insulin secretion pathways. Therefore, this study aimed to investigate the gene expression profiles and the antioxidant signalling pathways in response to CRPE in rat INS-1E cells. 

## 2. Results 

### 2.1. Cytotoxicity of Coloured-Rice Phenolic Extracts (CRPE) on INS-1E Cells

**Figure 1:**
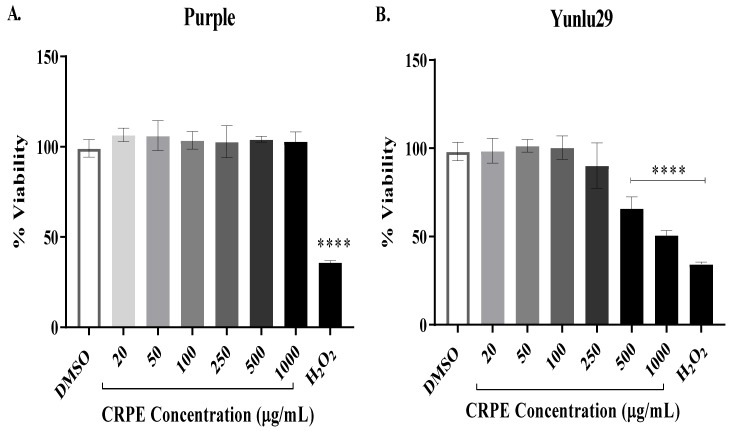
Cytotoxicity of coloured rice phenolic extracts (CRPE) on INS-1E cells. The cells were exposed to coloured rice extracts (purple [**A**] and Yunlu29 [**B**]) at various concentrations for 6 h, followed by resazurin red assay (*n* = 5). Asterisks indicate significant differences to the control group (DMSO), where, ****p < 0.0001; one-way ANOVA with turkey’s multiple comparison post hoc test. Data is presented as mean ± SEM. DMSO–-dimethyl sulfoxide; H_2_O_2_ –-Hydrogen peroxide.

The cytotoxic effect of coloured rice extracts on INS-1E cells was determined using resazurin red cytotoxic assay. Figure 1A demonstrates the purple variety extracts did not have any cytotoxic effect on INS-1E cells across all the tested concentrations. However, a significant (*p* < 0.0001) cytotoxic effect on INS-1E cells was observed at 500 and 1000 μg/mL for the Yunlu29 extracts (Figure 1B), reducing cell viability to 66% and 58% respectively post CRPE treatment when compared to the DMSO control group. There was no significant cytotoxic effect observed for the Yunlu29 extracts at 20, 50, 100, and 250 μg/mL. Therefore, CRPE concentrations of 50, 100 and 250 μg/mL were chosen for further experiments in this study.

### 2.2. Coloured Rice Phenolic Extracts Modulate the Expression of Genes Associated with β-cell Function 

To assess the gene modulating effects of CRPE on INS-1E cells under high (HGSC) and normal (NGC) glucose conditions, several β-cell function gene candidates (*Glut2*, *Sirt1*, *Tfam*, *Pdx-1* and *Ins1*) were selected. These genes regulate major pathways including glucose transport (*Glut2*), electron transport chain and mitochondrial ATP pathway (*Sirt1*), mitochondrial ATP/ADP ratio and mitochondrial DNA stability (*Tfam*), regulation of β-cell transcription factors (*Pdx-1*) and insulin biogenesis and exocytosis (*Ins1*). 

### 2.3. Expression of Glucose Transporter 2 (Glut2) Gene

**Figure 2:**
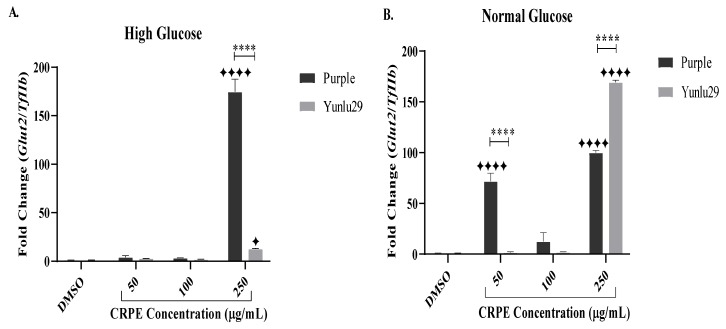
Expression of Glut2 in INS-1E cells. **A**) high glucose (25 mM). **B**) normal glucose (11.1 mM). *n* = 3. Stars indicate significant differences between extracts and DMSO, where ✦ *p* < 0.05, ✦✦✦✦*p* < 0.0001. Asterisks indicate significant differences between the CRPE extracts, where, ****p < 0.0001; two-way ANOVA with Turkey’s multiple comparison post hoc test. Data is presented as mean ± SEM. Glut2–glucose transporter 1; TfIIβ–transcription factor II β; INS-1E—Insulinoma cells; DMSO—dimethyl sulfoxide; CRPE—coloured rice phenolic extract.

For the HGSC, purple rice phenolic-rich extract significantly increased the expression of *Glut2* post CRPE treatment, when compared to the DMSO control (Figure 2A). Higher expression of *Glut2* was observed at 250 µg/mL compared to other doses of CRPE. Yunlu29 also significantly increased the expression of *Glut2* when compared to the control at 250 µg/mL. On comparing the two extracts, Purple extract displayed a more prominent (*p* < 0.0001) expression of *Glut2* than Yunlu29. 

Under NGC, a significant upregulation of *Glut2* was observed in purple rice extract when compared to control at 50 µg/mL and 250 µg/mL (Figure 2B). The red variety, Yunlu29, at 250 µg/mL significantly increased the expression of *Glut2*, when compared to the control. On comparing the two extracts, Purple exhibited a 65-fold change increase (*p* < 0.0001) in the expression of *Glut2* at 50 μg/mL than Yunlu29 extracts. However, at 250 μg/mL, Yunlu29 significantly (0.0001) upregulated the expression of *Glut2* by 70-fold compared to purple. 

### 2.4. Expression of Silent Mating Type Information Regulation 2 Homolog 1 (Sirt1) Gene

**Figure 3:**
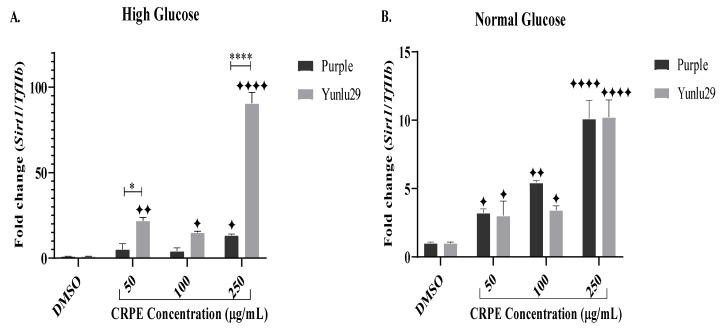
Expression of Sirt1 in INS-1E cells. **A**) high glucose (25 mM). **B**) normal glucose (11.1 mM). *n* = 3. Stars indicate significant differences between extracts and DMSO, where ✦ *p* < 0.05, ✦ ✦*p* < 0.01, ✦✦✦✦*p* < 0.0001. Asterisks indicate significant differences between the extracts, where *p < 0.05, ****p < 0.0001; two-way ANOVA with Turkey’s multiple comparison post hoc test. Data is presented as mean ± SEM. Sirt1—silent mating type information regulation 2 homolog 1; TfIIβ—transcription factor II β; INS-1E—Insulinoma cells; DMSO—dimethyl sulfoxide; CRPE—coloured rice phenolic extract.

Purple phenolic-rich extract markedly increased the expression of *Sirt1*, only at 250 µg/mL, compared to the control under HGSC (Figure 3A). Yunlu29 phenolic-rich extract significantly upregulated the expression of *Sirt1* across all the CRPE concentrations. Upregulation of *Sirt1* was greatest at 250 µg/mL when compared to the other doses of Yunlu29 phenolic extract. Comparing the two extracts, Yunlu29 increased the expression of *Sirt1* significantly at 50 (*p* < 0.05) and 250 µg/mL (*p* < 0.0001), respectively. 

Under NGC, a significant upregulation of *Sirt1* was observed post purple CRPE treatments compared to the control (Figure 3B). Yunlu29 displayed a similar upregulation of *Sirt1* at 50 and 100 µg/mL phenolic concentrations. To a similar extent, both phenolic-rich extracts displayed significant upregulation of *Sirt1* at 250 µg/mL.

### 2.5. Expression of Mitochondrial Transcription Factor A (Tfam) Gene

**Figure 4:**
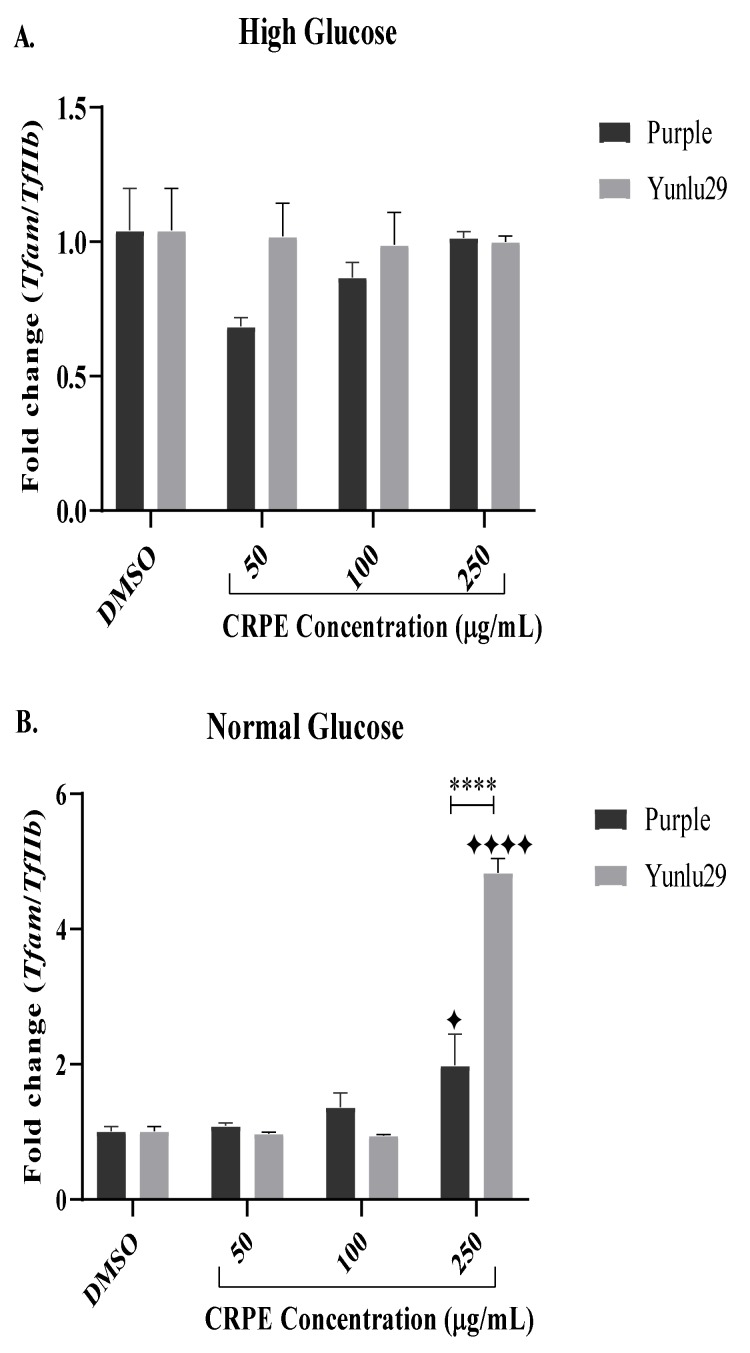
Expression of Tfam in INS-1E cells. **A**) high glucose (25 mM). **B**) normal glucose (11.1 mM). *n* = 3. Stars indicate significant differences between extracts and DMSO, where ✦ *p* < 0.05, ✦✦✦✦*p* < 0.0001. Asterisks indicate significant differences between the extracts, where ****p < 0.0001; two-way ANOVA with Turkey’s multiple comparison post hoc test. Data is presented as mean ± SEM. Tfam—mitochondrial transcription factor A; TfIIβ—transcription factor II β; INS-1E – Insulinoma cells; DMSO—dimethyl sulfoxide; CRPE—coloured rice phenolic extract.

Both purple and Yunlu29 CRPE displayed no significant effects on *Tfam* under HGSC (Figure 4A). Under NGC, however, both extracts significantly increased the expression of *Tfam* at 250 µg/mL when compared to the control group (Figure 4B). On comparing the two extracts, treatment with Yunlu29 phenolic-rich extract at 250 µg/mL displayed a three-fold increase in the expression of *Tfam* relative to purple.

### 2.6. Expression of Pancreatic and Duodenal Homeobox-1 (Pdx-1) Gene

**Figure 5:**
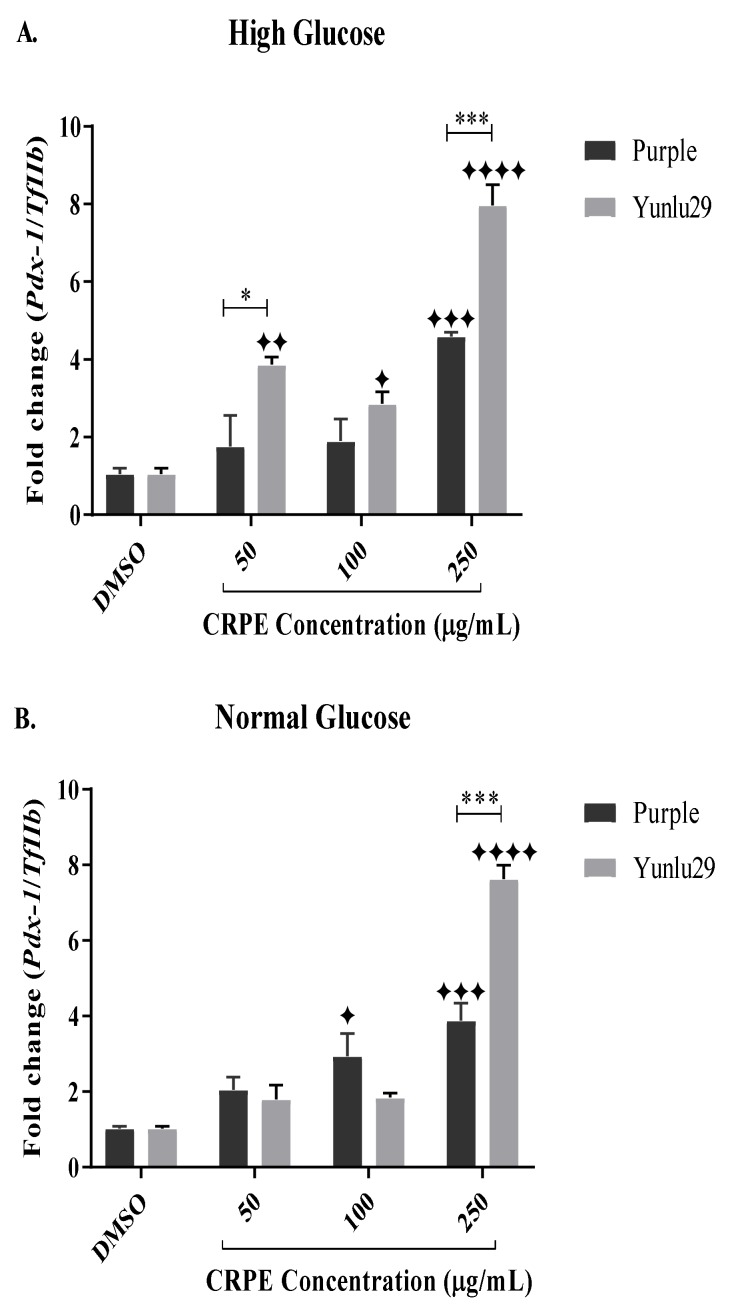
Expression of Pdx-1 in INS-1E cells. **A**) high glucose (25 mM). **B**) normal glucose (11.1 mM). *n* = 3. Stars indicate significant differences between extracts and DMSO, where ✦ *p* < 0.05, ✦ ✦*p* < 0.01, ✦✦✦*p* < 0.001, ✦✦✦✦*p* < 0.0001. Asterisks indicate significant differences between the extracts, where *p < 0.05, ***p < 0.001;two-way ANOVA with Turkey’s multiple comparison post hoc test. Data is presented as mean ± SEM. Pdx-1—pancreatic and duodenal homeobox-1; TfIIβ —transcription factor II β; INS-1E—Insulinoma cells; DMSO—dimethyl sulfoxide; CRPE—coloured rice phenolic extract.

Post treatment, both extracts exhibited significant upregulation of *Pdx-1* across all CRPE concentrations, when compared to the control under high glucose conditions (Figure 5A). Purple extract significantly increased the expression of *Pdx-1*, only at 250 µg/mL, compared to the control. Yunlu29 extract significantly upregulated the expression of *Pdx-1* at 50, 100 and 250 µg/mL. When comparing the two extracts, expression of *Pdx-1* was higher (*p* < 0.001) with Yunlu29 extract than the purple variety. 

In NGC (Figure 5B), purple extract significantly increased the expression of *Pdx-1* at 100 and 250 µg/mL when compared to the control. For Yunlu29, an increased expression of *Pdx-1* was displayed only at 250 µg/mL compared to the control. On comparing the two extracts, a significantly (*p* < 0.001) higher upregulation of *Pdx-1* was observed in Yunlu29 than purple at 250 µg/mL only. 

### 2.7. Expression of Insulin 1 (Ins1) Gene

**Figure 6:**
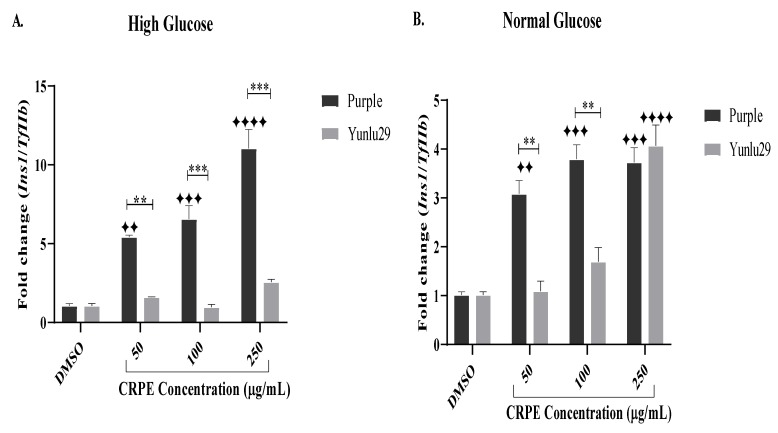
Expression of Ins1 gene on INS-1E cells. **A**) high glucose (25 mM). **B**) normal glucose (11.1 mM). *n* = 3. Stars indicate significant differences between extracts and DMSO, where ✦✦*p* < 0.01, ✦✦✦*p* < 0.001, ✦✦✦✦*p* < 0.0001. Asterisks indicate significant differences between the extracts, where **p < 0.01, ***p < 0.001; two-way ANOVA with Turkey’s multiple comparison post hoc test. Data is presented as mean ± SEM. Ins1—insulin 1; TfIIβ—transcription factor II β; INS-1E—Insulinoma cells; DMSO—dimethyl sulfoxide; CRPE—coloured rice phenolic extract.

Following 48 h exposure to HGSC, treatment with Purple rice extract exhibited a significant upregulation of *Ins1* across all the CRPE test concentrations when compared to the high glucose only treated cells (Figure 6A). When compared to Yunlu29, purple significantly (*p* < 0.01, *p* < 0.001, *p* < 0.001) upregulated the expression of *Ins1* at 50, 100 and 250 µg/mL, respectively. 

Under NGC, a significant upregulation of *Ins1* was observed with all concentrations of purple CRPE treatments when compared to the control (Figure 6B). Yunlu29 generated a significant increase in *Ins1* expression at 250 µg/mL. When comparing the two extracts, purple significantly (*p* < 0.01) upregulated the expression of *Ins1* relative to Yunlu29 at 50 and 100 µg/mL.

## 3. Discussion

Pancreatic β-cell dysfunction, a major cause of T2DM, is strongly associated with chronic high blood glucose levels. Chronic exposure of pancreatic β-cells to high glucose induces oxidative stress and decreases expression of β-cell function genes leading to an impaired insulin secretion [26]. The current investigation demonstrated that treatment of INS-1E cells with phenolic extracts from purple and Yunlu29 rice varieties increased the expression of key genes associated with β-cell function under both HGSC and NGC. 

Under HGSC, purple phenolic extracts significantly increased the expression of *Glut2* and *Ins1*, whereas Yunlu29 extracts significantly increased the expression of *Sirt1* and *Pdx-1*, however, neither variety affected *Tfam* expression. Under NGC, the purple variety had the greatest impact on expression of *Glut2* and *Ins1* at lower phenolic extract concentrations of 50 and, 50 and 100 µg/mL respectively. Yunlu29 variety markedly increased expression of *Glut2*, *Tfam* and *Pxd-1* at 250 µg/mL when compared to the purple variety. Both extracts increased the expression of *Ins1* and *Sirt1* to a similar degree (at 250 µg/mL) under NGC. 

The gene expression trends exhibited in this study are interesting when considering the phenolic composition of these rice varieties. The purple rice variety contained the highest total anthocyanin and proanthocyanidin content compared to the other varieties including Yunlu 29, with cyanidin-3-glucoside and peonidin-3-glucoside the most abundant anthocyanins with the highest antioxidant activities [27]. In contrast, Yunlu29 had the highest total phenolic content (235.50 ± 22.51 mg 100 g^−1^ gallic acid equivalent [GAE]) compared to the purple (172.40 ± 20.06 mg 100 g^−1^ GAE). The predominant phenolic acids were ferulic acid, o-coumaric acid, GA, syringic acid, flavanols, catechins and proanthocyanins [27,28]. Additionally, crude CRPEs were shown to contain various unidentified compounds that may have arrays of effects from antagonist to null to synergistic effects on expression of various genes and other cell processes [29]. The presence of these compounds in Yunlu29 extract was correlated with higher antioxidant activity [27], suggesting that the gene expression effects observed following treatment with Yunlu29 phenolic extracts may be due to synergistic effects of the mixture of these compounds. Although physiological concentrations of these compounds have not been determined, consumption of purple and Yulnu29 varieties increased antioxidant activities within 30 min and 1 h respectively in the obese cohort [30]. 

Addition of 250 µg/mL of purple rice extract to INS-1E cells significantly increased the expression of *Glut2* under HGSC. A previous study reported that treatment of INS-1 cells with 100 µM cyanidins, a major anthocyanin, upregulated the expression of *Glut2* and this correlated with increased insulin secretion and improved β-cell function [31]. In light of this finding, it is suggested that the increased expression of *Glut2* observed in this investigation may be associated with the high total anthocyanin content in the purple rice extracts, affecting some upstream glucose sensing pathways, which may inhibit the expression of *Glut2* under HGSC.

The present study also showed that supplementation of INS-1E with phenolic extract (250 µg/mL) from the red variety Yunlu29 increased the expression of *Glut2* ten-fold when compared to the control. In accordance with this result, treatment of diabetic rats with 50 mg/kg body weight (BW) ferulic acid, an abundant compound in Yunlu29, increased *Glut2* expression in hepatic tissues, thereby improving insulin sensitivity and glucose levels [32]. These findings indicate CRPE may increase *Glut2* expression by mitigating high glucose-induced oxidative stress on pancreatic β-cells, which may lead to increased insulin secretion and β-cell function. 

When considering the NGC, purple rice phenolic-rich extracts substantially increased *Glut2* expression at 50 and 250 µg/mL, however, there was a discontinuity of dose response in *Glut2* expression at 100 µg/mL (Figure 4B). An early study reported that polyphenols often display non-linear bioactivities by exhibiting a low dose stimulation and a high dose inhibition, creating a biphasic dose response [33]. This supports the notion that some polyphenols may have optimal concentrations where they structurally interact better with other cellular proteins, thereby increasing their efficacy [34]. Whether the results presented here reflect a biphasic effect is a question for further investigation. The Yunlu29 extract displayed a significant increase in *Glut2* expression 250 µg/mL when compared to the untreated control. A 70-fold change increase in *Glut2* expression by the Yunlu29 extract may indicate a synergistic effect relative to the higher total phenolic content in Yunlu29, when compared to Purple extracts [27]. 

Although studies focusing on the modulating effects of CRPEs on β-cell function genes, especially genes associated with mitochondrial function, are not available, other phenolic compounds found in coloured rice varieties have been shown to modulate β-cell function genes. A study involving diabetic rats revealed that treatment with 50 mg/kg BW of proanthocyanidins, which were a major component in purple extracts increased expression of *Sirt1* dose-dependently and was correlated with elevated NAD^+^ in hepatic tissues thereby increasing mitochondrial ATP synthesis and β-cell function [35]. In the present study, we observed a significant decrease in *Sirt1* expression under HGSC, but pre-treatment with CRPE (purple) significantly increased the expression of *Sirt1* in INS-1E cells, which is suggestive of free radical scavenging properties. 

*Sirt1* gene expression increases elicited by the addition of phenolic flavanols has been linked to improved β-cell function [25]. Studies of mitochondrial function in pancreatic β-cell-derived INS-1 832/13 demonstrated supplementation with 25 µg/mL catechin extract, a major compound in Yunlu29, increased the expression of *Sirt1* down-stream genes concomitantly improving mitochondrial ATP production, reduced oxidative stress and high glucose levels, and improved β-cell function [25,36]. Our study has shown that exposure of INS-1E cells to Yunlu29 extracts (250 µg/mL) significantly upregulated *Sirt1* post HGSC. These results propose antioxidant mechanisms of the Yunlu29 CRPE evoking gene upregulation and may potentially restore β-cell function.

It is interesting to note that post HGSC, treatment with Yunlu29 extract increased the expression of *Sirt1* 60-fold when compared to the Purple extract. The distinct expression pattern changes in response to each extract addition may indicate structural aspects play a role in their bioactivities. Catechins, a major constituent of Yulun29, possess hydroxyl (3-OH) groups, which render them capable of scavenging free radicals by terminating the cycle of new free radical formation during oxidative stress conditions [37]. Based on this, it was postulated that supplementation with a monomeric catechin-rich cocoa fraction preserved mitochondrial membrane integrity, reduced ROS production and improved β-cell function [37]. 

Furthermore, we observed that treatment with CRPEs increased *Sirt1* expression under NGC. As previously reported, supplementation with phenolic-rich resveratrol (30 mg/kg BW) increased *Sirt1* expression in male rats, which increased insulin secretion and β-cell function [38]. As such, the current study indicates that exposure to CRPEs may increase insulin secretion and thus enhance normal β-cell function making CRPEs potential dietary supplements to prevent the development of β-cell dysfunction. 

*Tfam* is a transcription factor that maintains mitochondrial DNA stability, and regulates the electron transport chain and insulin exocytosis events. Dietary polyphenols have been shown to increase expression of this gene through their antioxidant capability. In the current investigation, however, CRPEs from both varieties exhibited no modulating effects on *Tfam* under HGSC, suggesting that the expression of *Tfam* might be affected by different mechanisms under oxidative stress and that CRPEs perhaps modulate a different pathway. Further, crude CRPEs contain unidentified compounds, and these unknown compounds may have some antagonistic activities under HGSC making further studies warranted. 

In contrast, CRPEs from both varieties significantly increased *Tfam* expression under NGC. This is consistent with other reports where treatment of INS-1E with resveratrol increased *Tfam* expression under NGC. The resulting increased ATP/ADP ratio and mtDNA stability was correlated with improved insulin secretion and β-cell function in INS-1E cells [20]. As a multi-functional DNA binding protein, *Tfam* may have similar transcriptional activities with *Sirt1* and *Pdx-1* in insulin secretion pathways [39]. Therefore, the three-fold change in *Tfam* expression by Yunlu29 extract compared to purple perhaps relates to the catechin activities, which may maintain β-cell function, thus preventing the development of T2DM.

Pancreatic and duodenal homeobox-1 plays diverse transcription roles in β-cell function, but its deficiency has been associated with mitochondrial dysfunction and educed insulin secretion, some of the defining factors of β-cell dysfunction [40]. The current study demonstrated that purple CRPE increased the expression of *Pdx-1* markedly at concentrations of 250 μg/mL under HGSC. Treatment of INS-1 cells with C3G-rich extract (5 μM) significantly increased the expression of *Pdx-1* post exposure to H_2_O_2_ (1,200 μM), which consequently reduced mitochondrial ROS, enhanced antioxidant capacity and elevated insulin secretion; collectively leading to an improved β-cell function [23]. These results are suggestive of a cytoprotective effect of purple CRPE on pancreatic β-cells under oxidative stress.

Yunlu29 CRPE increased the expression of *Pdx-1* under HGSC. Consistent with previous reports, treatment of pancreatic β-cells (RINm5F) with gallic acid (10µM) increased the expression of *Pdx-1* under high glucose (25 mM) conditions by decreasing Caspase-3 and Nf-kB signalling, which consequently increased insulin secretion and β-cell function [24]. This suggests the eight-fold change of *Pdx-1* expression by the Yunlu29 CRPE observed in the current study may relate to a higher gallic acid content in the Yunlu29 extracts inhibiting the activation of some apoptotic and inflammatory pathways under HGSC. 

Yunlu29 potentiated a 3.5-fold change upregulation of *Pdx-1* relative to purple. Raoet al. [27] found Yunlu29 possessed the highest total phenolic content and argued this was directly proportional to its higher antioxidant activities compared to purple. As such it is possible that the increased expression of *Pdx-1* in response to Yunlu29 phenolic extract addition may be due to the presence of various phenolic compounds affecting a synergistic antioxidant response. 

Under low glucose conditions, it is believed that *Pdx-1* is mainly localised in the nuclear periphery, where it’s linked with histone deacetylase-1 and −2, thus reducing its influence via transcriptional events on insulin secretion mechanisms [41]. This study showed that CRPEs significantly increased *Pdx-1* expression under NGC. Supplementation of INS-1E cells with anthocyanin extracts (0.125–1 mg/mL) elevated the expression of free fatty acid receptor 1 (*FFAR1*), a *Pdx-1*-like gene that stimulates glucose-dependent insulin secretion response in pancreatic β-cells, at baseline glucose conditions [42]. Additionally, ferulic acid (0.5 g/kg BW) addition reduced blood glucose levels, increased insulin secretion and improved β-cell function in male mice [43]. These results suggest a key role for CRPEs in enhancing β-cell function under NGC. Therefore, *Pdx-1* expression increases in response to CRPEs under NGC may indicate glucose stimulating-like insulin secreting properties. 

To ensure proper pancreatic β-cells function, regulation of *Ins1* is crucial for the synthesis and secretion of insulin to maintain normal blood glucose concentrations. The present study showed that *Ins1* was severely reduced in HGSC, however, pre-treatment with purple CRPE dose-dependently increased the expression of *Ins1*. It was previously demonstrated that treatment of INS-1E with anthocyanin extracts restored the insulin pool and influenced the opening of voltage gated Ca^2+^ channels, which led to an increased insulin-membrane fusion and exocytosis [44]. This suggests that the purple CRPE may be targeting voltage gated Ca^2+^ channel pathways to increase the expression of *Ins1*. The inactivity observed in response to Yunlu29 indicates that the phenolic compounds in the Yunlu29 extracts may be affecting different pathways from insulin biosynthesis and exocytosis mechanisms.

An early study showed that treatment of pancreatic β-cells under 4 mM glucose with *Cornus* fruit extract containing various anthocyanins compounds including pelargonidin, pelargonidin/peonidin-3-glucoside and delphinidin-3-glucoside exhibited a 1.4-fold increase in insulin secretion in the increasing order of delphinidin-3-glucoside, cyanidin-3-glucoside and pelargonidin-3-galactoside [45]. This indicates that the insulin secretion enhancing activity of anthocyanins may be attributed to the number of hydroxyl groups on the B-ring. Our investigation revealed that supplementation with purple CRPE upregulated *Ins1* under NGC. A similar finding reported pre-treatment of INS-1E with anthocyanin-rich berry mix extracts (50 µM) increased the expression of *Ins1*, which improved insulin secretion and β-cell function even in the absence of glucose [46]. Taken together, these results suggest that the glycosidic side chains of anthocyanins in the purple extract may assume glucose structural activity, thereby constituting a glucose mimetic effect. This may support the notion that CRPEs may promote insulin secretion through nuclear activation required for the upregulation of β-cells function genes under NGC.

Purple CRPE exhibited a significant impact on *Ins1* expression at lower concentrations while Yunlu29 exhibited no modulatory effect under these concentrations. This may be explained by the presence of more hydroxyl groups coupled with the higher total anthocyanin content in the purple CRPE. However, both extracts increased the expression of *Ins1* to a similar degree at 250 µg/mL, suggesting that the higher total phenolic content in the Yunlu29 extracts may have been equalised by the higher number of hydroxyl groups in the Purple CRPE. Pancreatic β-cell function is defined by the amount of insulin secreted. Therefore, GSIS, an assay not performed in this study, is required to determine the level of insulin secreted in relation to these gene expression changes. Further, protein content, gene translocation and protein phosphorylation studies are warranted to ascertain whether these gene expression changes are manifested in downstream biological events. 

## 4. Materials and Methods

### 4.1. Materials

Unless otherwise stated, reagents and chemicals were purchased from Sigma-Aldrich (St Louis, Missouri, USA), Chem Supply Pty Ltd (Port Adelaide, South Australia, Australia), Promega Corporation (Madison, WI, USA) and Bio-Rad (Hercules, CA, USA.) 

### 4.2. Coloured Rice Sampling and Phenolic Extraction

Coloured rice (*Oryza sativa*) cultivars, Purple and Yunlu29 used in this study were obtained as part of the field trials performed in Mackay, Queensland by the New South Wales Department of Primary Industries and Rice Research Australia Pty Ltd. The samples were subjected to the same routine growing and post-harvest conditions before storing at 4 °C. The two varieties purple and red Yunlu29 were selected for this study based on their antioxidant activities, and phenolic extraction as previously described [27]. Purple extract contains mainly cyanidin-3-glucoside and peonidin-3-glucoside, and has the highest anthocyanins and proanthocyanins content. Yunlu29 predominantly possesses catechins and other phenolic acids, and has the highest total phenolic content associated with the highest antioxidant activities [27].

### 4.3. Cells and Cell Culture Conditions

The clonal rat β-cell line (INS-1E) was a kind gift from Dr Kathryn Aston-Mourney, Head of Islet Biology Laboratory at Deakin University. INS-1E cells were grown in RPMI1640 complete media supplemented with 2 mM L-glutamine, 1 mM sodium pyruvate, 10 mM HEPES, 0.05 mM β-mercaptoethanol, 10% FBS and 10,000 U/mL penicillin and 10 mg/mL streptomycin. The cells were maintained in a humidified atmosphere containing 5% CO_2_ at 37 °C, with the complete growth media changed every 48 h until confluence. All experiments were carried out using INS-1E passages between 37 and 40. 

### 4.4. Cytotoxicity Assay

The cytotoxic effect of CRPE on INS-1E cells was performed using resazurin red cytotoxicity assay. INS-1E cells were seeded at a density of 4 x 10^4^ cells per well into 96-well plates and incubated for 24 h in the complete growth media. After reaching confluence, the cells were treated with CRPE supplemented to the complete growth media at varying concentrations of 20, 50, 100, 250, 500 and 1000 μg/mL for 6 h. After treatment, CRPE supplemented media was removed and 200 µL of 14 mg/mL resazurin dye supplemented media was added to each well, followed by 4 h incubation at 37 °C in 5% CO_2_. Subsequently, 150 μl of the resazurin dye supplemented media was removed from each well and transferred to a new 96-well plate. The absorbance was measured at dual wavelengths (570 and 600 nm) using a microplate reader (FLUOstar Omega microplate reader, BMG Labtech, Offenburg, Germany). CRPE supplemented expansion media was used as the blank for each tested concentration. A media containing 0.25% dimethyl sulfoxide (DMSO) was used as the negative control. The percentage of cell viability in relation to the reduction of the dye was calculated as previously described [47]. 

### 4.5. Experimental Design and High Glucose Stress Induction

Two different glucose conditions were used; 1) high glucose (25 mM)-induced stress condition (HGSC) to induce glucotoxic stress and; 2) normal glucose (11.1 mM) condition (NGC) to represent a normal β-cell function [48,49]. INS-1E cells were seeded onto six-well plates at a density of 4 x 10^5^ cells per well and expanded in culture media and conditions as described in Section 4.3. After 24 h incubation, the media was removed, and the cells were washed with phosphate buffered saline (PBS). For the high glucose conditions, INS-1E cells were further subjected to 48 h incubation in 25 mM glucose supplemented growth media. The cells were then supplemented with CRPE at 50, 100 and 250 µg/mL, for 6 h. The wells treated with 0.25% DMSO served as the negative controls. All experiments were performed in triplicates.

### 4.6. Ribonucleic Acid (RNA) Extraction 

Post CRPE treatment, INS-1E cells were washed with ice-cold PBS. Total RNA extraction was performed using SV Total RNA Isolation System (Promega, Madison, WI, USA) following the manufacturer’s instructions with slight modifications. Briefly, 175 µL lysis buffer containing β-mercaptoethanol was used to lyse the cells. The lysate was then mixed with 350 µL RNA dilution buffer and incubated at 70 °C for 3 min. After centrifugation, the supernatant was mixed with 200 µL 95% ethanol and placed into the spin column assembly for DNase digestion. Following washing steps using RNA wash buffer, RNA was eluted in 35 µL nuclease-free water and stored at −80 °C until further use. 

### 4.7. Complementary Deoxyribonucleic Acid (cDNA) Synthesis

The quality of RNA was analysed using a NanoDrop™ 2000c Spectrophotometer (Thermo Fisher Scientific, Waltham, MA, USA). Five hundred nanograms of total RNA template was used to generate cDNA using GoScript™ Reverse Transcription system (Promega, Madison, WI, USA) according to the manufacturer’s instructions. Briefly, cDNA synthesis was initiated by incubating the RNA with 1 µL of oligo (dT)_15_ at 70 °C for 5 min. The reverse transcription reaction mix containing GoScript™ 5× reaction buffer, MgCl_2_ (final concentration 1.5 mM), PCR nucleotide mix (final concentration 0.5 mM each dNTP), recombinant RNAsin^®^ ribonuclease inhibitor (optional), GoScript™ reverse transcriptase was then added. Nuclease-free water was added to bring the final volume to 20 µL. The amplification cycles involved a 5 min annealing step at 25 °C followed by extension at 42 °C for 1 h. The cDNA synthesis was completed by the inactivation of reverse transcriptase at 70 °C for 15 min and the samples were stored at −20 °C until further use.

### 4.8. Quantitative Polymerase Chain Reaction (qPCR)

Quantification of gene expression was performed on CFX96 Touch™ Real-Time PCR Detection System (Bio-Rad) using SSoAdvanced Universal SYBR Green Supermix (Bio-Rad Laboratories, CA, USA) following the manufacturer’s instructions. Briefly, a single reaction mix consisted of 5 µL of Supermix (Bio-rad, CA, USA), 1 µM of forward and reverse primers and 1 µL of 1:5 diluted cDNA. The primers used for qPCR study (Table 1) were adopted from previous work by Vetterliet al. [20]. Transcription factor II β (*TfIIb*) was used as the house keeping gene for gene expression normalisation. Gene expression analysis was performed using Q-gene software as described by Muller et al. [50].

### 4.9. Statistical Analysis

Data analysis were performed using GraphPad Prism 8 software (Graphpad Software Inc, San Diago, CA, USA). The data for cytotoxicity assay were analysed using one-way analysis of variance (ANOVA). The fold change differences in gene expression were analysed using two-way ANOVA followed by Tukey’s multiple comparisons test. Statistically significant difference was determined at the level of *p* < 0.05. Data are presented as mean ± standard error of mean (SEM) of three independent experiments.

## 5. Conclusions

This study demonstrated that coloured rice phenolic extracts derived from purple and Yunlu29 varieties, increased the expression of β-cells function genes (*Glut2*, *Sirt1*, *Pdx-1* and *Ins1*) under both high glucose and normal glucose conditions. Purple phenolic-rich extract exhibited greater increases in *Glut2* and *Ins1* expression under HGSC, possibly due to the high content of anthocyanin and proanthocyanin compounds. Overall, Yunlu29 CRPE displayed the greatest increase in expression of the β-cell function genes, potentially due to its high content of total phenolic compounds linked to high antioxidant activities. Further in vitro and in vivo studies involving GSIS assays and post-transcriptional analysis are warranted to investigate the insulin secretion mechanisms in relation to the gene expression profiles of CRPE. Future studies may also involve antioxidant signaling mechanisms in relation to pancreatic β-cell insulin secretion. If the results of the current study translate into insulin secretion and possibly clinical relevance, it may mean that CRPE can stimulate insulin secretion both in low and chronic glucose conditions, and thus may provide a therapeutic capability.

## Figures and Tables

**Table 1:** List of the genes and primers used for qPCR.

Genes	Origin	Sense (5’-3’)	Antisense (3’-5’)
***Glut2***	Rat	TCAGCCAGCCTGTGTATGCA	TCCACAAGCAGCACAGAGACA
***Sirt1***	Rat	CAGTGTCATGGTTCCTTTGC	CACCGAGGAACTACCTGA T
***Tfam***	Rat	GGGAAGAGCAAATGGCTGAA	TCACACTGCGACGGATGA GA
***Pdx-1***	Rat	CCGCGTTCATCTCCCTTT C	CTCCTGCCCACTGGCTTT T
***Ins1***	Rat	TGCTCACCCGCGACCTT	GTTCATATGCACCACTGGACTGAA
***TfIIb***	Rat	GTTCTGCTCCAACCTTTGCCT	TGTGTAGCTGCCATCTGCACT T

## Abbreviations

ATP	Adenosine triphosphate
CRPE	Coloured rice phenolic extract
DMSO	Dimethyl sulfoxide
Glut2	Glucose transporter 2
GSIS	Glucose stimulated insulin secretion
HGSC	High glucose-induced stress condition
mtDNA	Mitochondrial DNA
NAD	Nicotinamide adenine dinucleotide
NF-kB	Nuclear factor kappa light chain enhancer of activated B cells
NGC	Normal glucose condition
Pdx-1	Pancreatic and duodenal homeobox-1
RNA	Ribonucleic acid
ROS	Reactive oxygen species
Sirt1	Silent mating type information regulation 2 homolog 1
T2DM	Type 2 diabetes mellitus
Tfam	Mitochondrial transcription factor A
